# Implications of Retrosternal Extension on Postoperative Serum Calcium Levels Following Total Thyroidectomy: A Retrospective Study

**DOI:** 10.7759/cureus.73050

**Published:** 2024-11-05

**Authors:** Hithyshree Nagaraj, S.M. Azeem Mohiyuddin, Sagayaraj A, Kouser M, Ravindra P Deo, Susanna Theophilus Yesupatham, Kalyani Raju, Anil K Sakalecha

**Affiliations:** 1 Otorhinolaryngology-Head and Neck Surgery, Sri Devaraj Urs Medical College, Kolar, IND; 2 Otolaryngology, Sri Devaraj Urs Medical College, Kolar, IND; 3 Biochemistry, Sri Devaraj Urs Medical College, Kolar, IND; 4 Pathology, Sri Devaraj Urs Medical College, Kolar, IND; 5 Radiodiagnosis, Sri Devaraj Urs Medical College, Kolar, IND

**Keywords:** central compartment clearance, postoperative hypocalcemia, retrosternal extension of thyroid, thyroid malignancy, total thyroidectomy

## Abstract

Introduction: Total thyroidectomy is a common surgery in otorhinolaryngology, with hypocalcemia being a potential complication, either transient or permanent. Calcium plays a critical role in many physiological processes, including nerve transmission, cardiac function, and muscle activity. Postoperative hypocalcemia can occur within 48 hours or be delayed up to four days. Risk factors include thyroid size, vascularity, retrosternal extension, and surgical extent. Timely treatment is essential, especially in acute cases, to avoid long-term complications. The objective of the study is to evaluate the impact of retrosternal extension and central compartment clearance on postoperative hypocalcemia in patients undergoing total thyroidectomy and the duration and severity of hypocalcemia.

Methods: A retrospective analysis was conducted on patients who underwent total thyroidectomy at a tertiary rural hospital from January 2016 to June 2024. Patients were categorized into two groups: those with retrosternal extension and/or central compartment clearance and those without. Postoperative serum calcium levels were documented over four days post-surgery, and the incidence and duration of hypocalcemia were compared between the groups.

Results: Out of 69 patients, 21 (30.4%) developed hypocalcemia postoperatively. Patients with retrosternal extension had a higher incidence of hypocalcemia (odds ratio = 3.58) compared to those without. Additionally, patients with central compartment clearance showed a higher risk of early postoperative hypocalcemia. The severity of hypocalcemia was greater in patients with malignancy and more extensive surgical procedures. Recovery time varied, with some patients requiring long-term calcium supplementation beyond one year.

Conclusion: Retrosternal extension and central compartment clearance significantly increase the risk of postoperative hypocalcemia. Although not statistically significant, the trends suggest a need for careful surgical techniques and rigorous postoperative calcium management to prevent prolonged hypocalcemia. Further prospective studies are recommended to confirm these findings and improve postoperative care strategies.

## Introduction

Total thyroidectomy is a frequently done surgery in the Department of Otorhinolaryngology. Hypocalcemia is one of the complications of total thyroidectomy, which can be transient (3-30%) or permanent (0.5-10.6%) [1]. Calcium is a key intracellular messenger and co-factor for various enzymes [2,3]. Ionized calcium, the physiologically active moiety, plays an essential role in many vital physiologic processes, including nerve impulse initiation and neurotransmission, cardiac conduction and contractility, blood coagulation, hormonal secretion, bone formation, skeletal and smooth muscle function, and a host of other crucial and diverse physiological functions [4-7].

Hypocalcemia can present as an incidental asymptomatic laboratory finding or as a severe, life-threatening condition and also increases the duration of hospitalisation [8,9]. Distinguishing acute from chronic hypocalcemia and asymptomatic from severely symptomatic hypocalcemia is critical for determining appropriate therapy. When acute hypocalcemia occurs, rapid treatment is mandatory. In contrast, chronic hypocalcemia may be well tolerated, but treatment is necessary to prevent long-term complications [10,11]. Clinically significant hypocalcemia may occur within 48 hours after thyroidectomy. However, latent hypocalcemia may be delayed up to four days after the surgery. Persistent hypocalcemia can result in chronic hypocalcemia.

The vascular supply for the parathyroid glands, which regulates parathyroid hormone (PTH) and, consequently, serum calcium levels, primarily arises from the intracapsular branches of the inferior thyroid artery, accounting for 60-80% of parathyroid vascularity. Vascular compromise due to cautery burn or ligature of the inferior thyroid artery proximally results in inadequate blood supply to the parathyroid gland and thereby results in hypocalcemia [12]. Since vascularity is crucial, central compartment clearance and retrosternal extension quite often make resection difficult and can result in either avulsion of the parathyroid gland or injury to its vascularity.

Risk factors for postoperative hypocalcemia following total thyroidectomy include thyroid gland size, vascularity of the gland, retrosternal extension of the thyroid, type of thyroid disorder, and extent of surgery [13]. Therefore, we performed a retrospective analysis that aims to compare hypocalcemia following total thyroidectomy with or without retrosternal extension and with or without central compartment clearance. Understanding these differences can aid in optimizing surgical techniques and postoperative management strategies to minimize complications and improve patient outcomes.

## Materials and methods

Source of data

All patients who underwent total thyroidectomy in the Department of Otorhinolaryngology and Head and Neck Surgery at a tertiary care rural hospital, meeting the inclusion criteria and screened against the exclusion criteria, were included in the study.

Inclusion criteria

All patients aged between 18 and 65 years who underwent total thyroidectomy in the Department of Otorhinolaryngology and Head and Neck Surgery at a tertiary care rural hospital were included.

Exclusion criteria

Patients who underwent completion total thyroidectomy following hemithyroidectomy performed elsewhere, patients with second primary tumours or recurrent tumours, patients with parathyroid adenoma or hyperparathyroidism prior to surgery and those with pre-existing severe osteoporosis and mal-absorption syndromes were excluded.

Methodology

This retrospective observational study was done using case records of patients who underwent total thyroidectomy with or without retrosternal extension and with or without central compartment clearance from January 2016 to June 2024. The study was started after approval from the Central Ethics Committee (SDUAHER/KLR/R&D/CEC/S/PG/38/2024-25). Consent was obtained from patient/patient attenders to review their medical records. Relevant data including demographic details of the patients who underwent total thyroidectomy was collected for the study from the medical records department. The size of the thyroid gland, its extent and any retrosternal extension, details regarding the tumour (benign or malignant), and the type of surgery performed - total thyroidectomy or total thyroidectomy with central compartment clearance - were documented. Preoperative and postoperative serum calcium levels, onset of hypocalcemia, intervention done for correction of hypocalcemia and duration of calcium supplements received were documented. All patients were followed up (mean follow-up of 1.2 years) and complications if any, after receiving long-term calcium supplements were analysed and documented.

Statistical methods

Data was entered using Microsoft Excel (Microsoft® Corp., Redmond, WA, USA) and analysed using the Statistical Package for the Social Sciences (IBM SPSS Statistics for Windows, IBM Corp., Version 23.0, Armonk, NY). The odds ratio was calculated by the log regression test and this ratio of various factors was determined in all the cases. It was further assessed using parametric Pearson and non-parametric Spearman rank correlation. The comparison of categorical data between groups was performed using the Chi-square test and Fisher’s exact test. The significance level was set at 5%.

## Results

The study involved 69 patients with an age range of 20 to 65 years, and the mean age was approximately 41 years, 33.9% were between 20 and 30 years, 19.6% between 31 and 40, 19.6% between 41 and 50 and 26.8% were between 50 and 65 years. Sixty-three patients were females and six were males.

Table 1 shows histopathological diagnosis, surgery performed and incidence of hypocalcemia. The most common histological diagnosis from the specimen was benign multinodular goitre (36.2%), followed by papillary carcinoma (28.9%) and adenomatous hyperplasia of the thyroid with nodules (13.04%). Less common cases include Hurthle cell carcinoma (7.2%), lymphocytic thyroiditis (8.6%), follicular neoplasm (2.8%) and medullary carcinoma (2.8%).

**Table 1:** Distribution of thyroid disorders, surgical interventions and hypocalcemia incidence among 69 patients.

Characteristics		Number of Patients	Percentage (%)
Diagnosis	Follicular neoplasm	2	2.8
	Hurtle cell carcinoma	5	7.2
	Lymphocytic thyroiditis	6	8.6
	Medullary carcinoma	2	2.8
	Benign multinodular goitre	25	36.2
	Adenomatous hyperplasia of thyroid with nodules	9	13.04
	Papillary carcinoma	20	28.9
Surgery	Total thyroidectomy	40	57.9
	Total thyroidectomy + central compartment clearance	29	42.1
Retrosternal extension	Present	28	40.57
	Absent	41	59.42
Patients with hypocalcemia	No	48	69.5
	Yes	21	30.4

**Figure 1:**
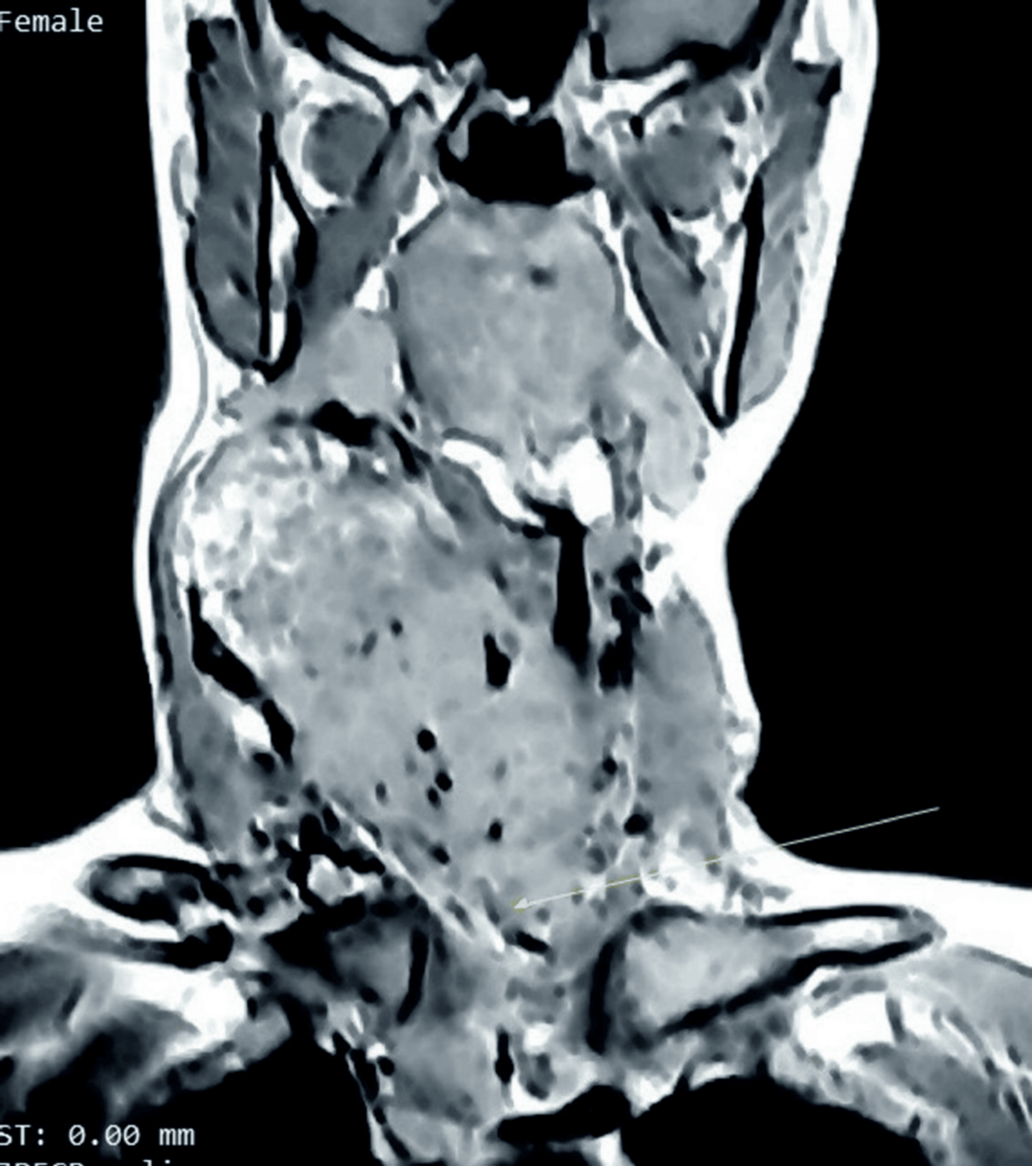
MRI of the neck in a 35-year-old female showing an enlarged thyroid gland with retrosternal extension. The yellow arrow indicating the retrosternal extension.

 Figure 1 shows an MRI of the neck of a 35-year-old lady with retrosternal extension of thyroid swelling.

**Figure 2:**
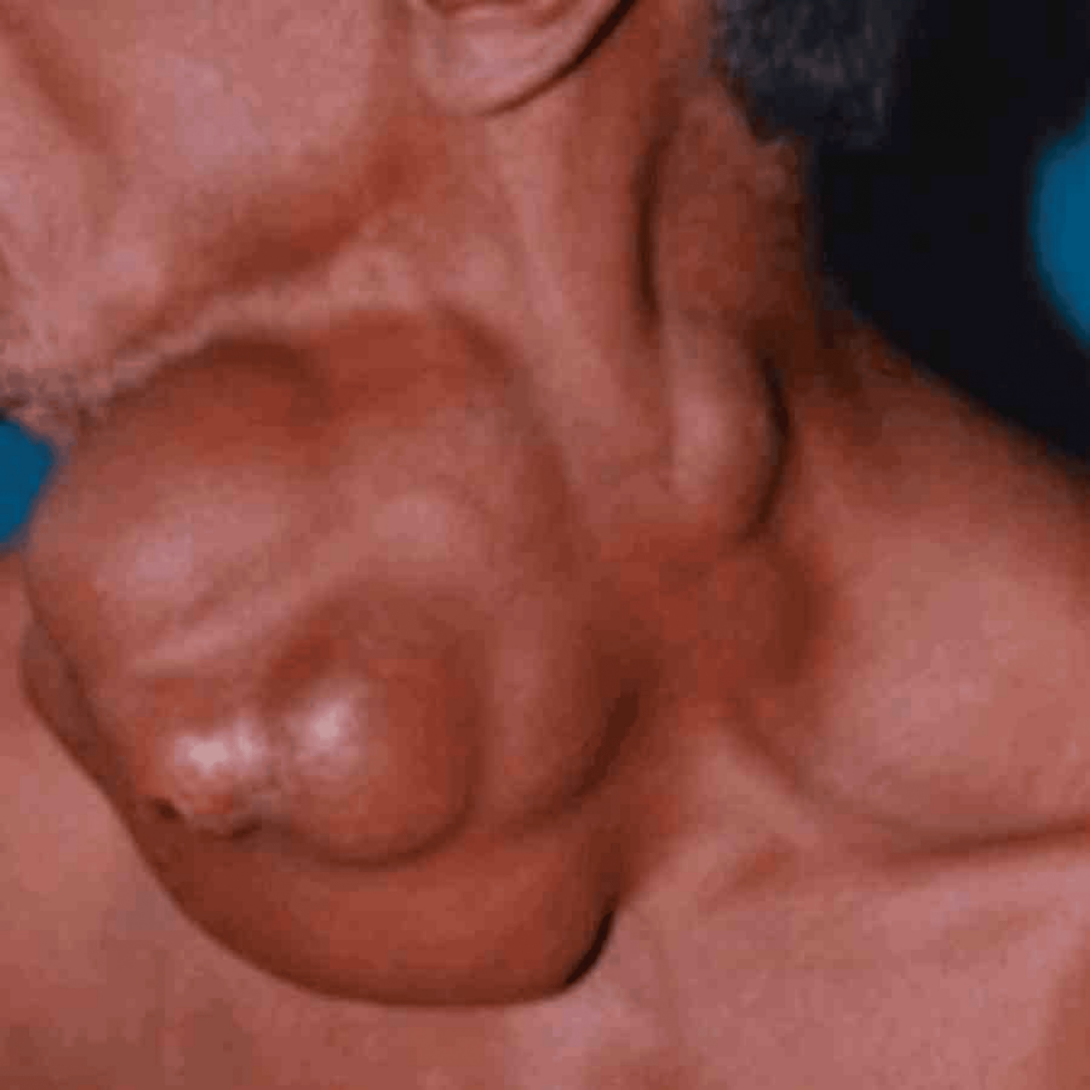
A 52-year-old male diagnosed with papillary thyroid carcinoma.

Figure 2 shows a case of papillary carcinoma thyroid with retrosternal extension and cervical lymph node metastasis.

Sixty-three per cent of the cases (44 patients) had no significant lymphadenopathy. Four cases presented with lymph nodes in levels II, III, IV, and the ipsilateral central compartment. Two cases had prominent lymph nodes in bilateral level III, and three had prominent nodes in bilateral level II. Two cases showed nodes in the central compartment and mediastinal lymph nodes. Four cases had multiple nodes in the central compartment, five had lymph nodes in bilateral levels II, III, and IV, and five patients had ipsilateral level III and IV lymph nodes with loss of fatty hila.

Table 2 presents a comparison of the onset of hypocalcemia in patients who underwent two different surgical procedures: total thyroidectomy and total thyroidectomy with central compartment clearance. Central compartment clearance is associated with a higher risk (odds ratio = 2.43) of hypocalcemia especially in the immediate postoperative period.

**Table 2:** Comparison of onset of hypocalcemia in patients following total thyroidectomy and total thyroidectomy with central compartment clearance. Statistical test: Chi-square test The reference range for serum calcium levels is 8.5 to 10.5 mg/dL [14].

Time point	Surgery				p-value
	Total thyroidectomy (n= 40)		Total thyroidectomy + central compartment clearance (n=29)		
	Patients with hypocalcemia	Serum calcium levels (mg/dL)	Patients with hypocalcemia	Serum calcium levels (mg/dL)	
Pre-op	0		1	8.3	-
Day 1	2	8.3	4	7.8	0.2201
				8.1	
		8.1		8	
				7.2	
Day 2	2	8.3	3	8	0.366
				8.2	
		8.1		6.7	
Day 3	2	8.2	2	8.2	0.62
		8.3		8.3	
Day 4	3	8.4	2	8.3	0.96
		8.2		8.2	
		8.2			

The odds of hypocalcemia are higher (odds ratio = 1.55) in patients with malignancy compared to those with benign pathology.

Table 3 compares the occurrence of hypocalcemia between patients with and without retrosternal extension at different time points. Retrosternal extensions have a higher risk of developing hypocalcemia in the early postoperative period compared to those without.

**Table 3:** Comparison of onset of hypocalcemia in patients with and without retrosternal extension. Statistical test: Chi-square test The reference range for serum calcium levels is 8.5 to 10.5 mg/dL [14].

Day	Retrosternal extension					p-value
	Present (n=28)			Absent (n=41)		
	Number of cases of hypocalcemia	Serum calcium levels (mg/dL)	Number of cases of hypocalcemia		Serum calcium levels (mg/dL)	
Pre-op	1	8.3	0		-	
Day 1	4	8	2		8.1	0.20
		8.3				
		7.2			8.1	
		7.8				
Day 2	3	8	2		8.2	0.35
		8.1			8.3	
		6.7				
Day 3	2	8.3	2		8.2	0.62
		8.2			8.3	
Day 4	3	8.3	2		8.2	0.32
		8.2			8.4	
		8.2				

**Figure 3:**
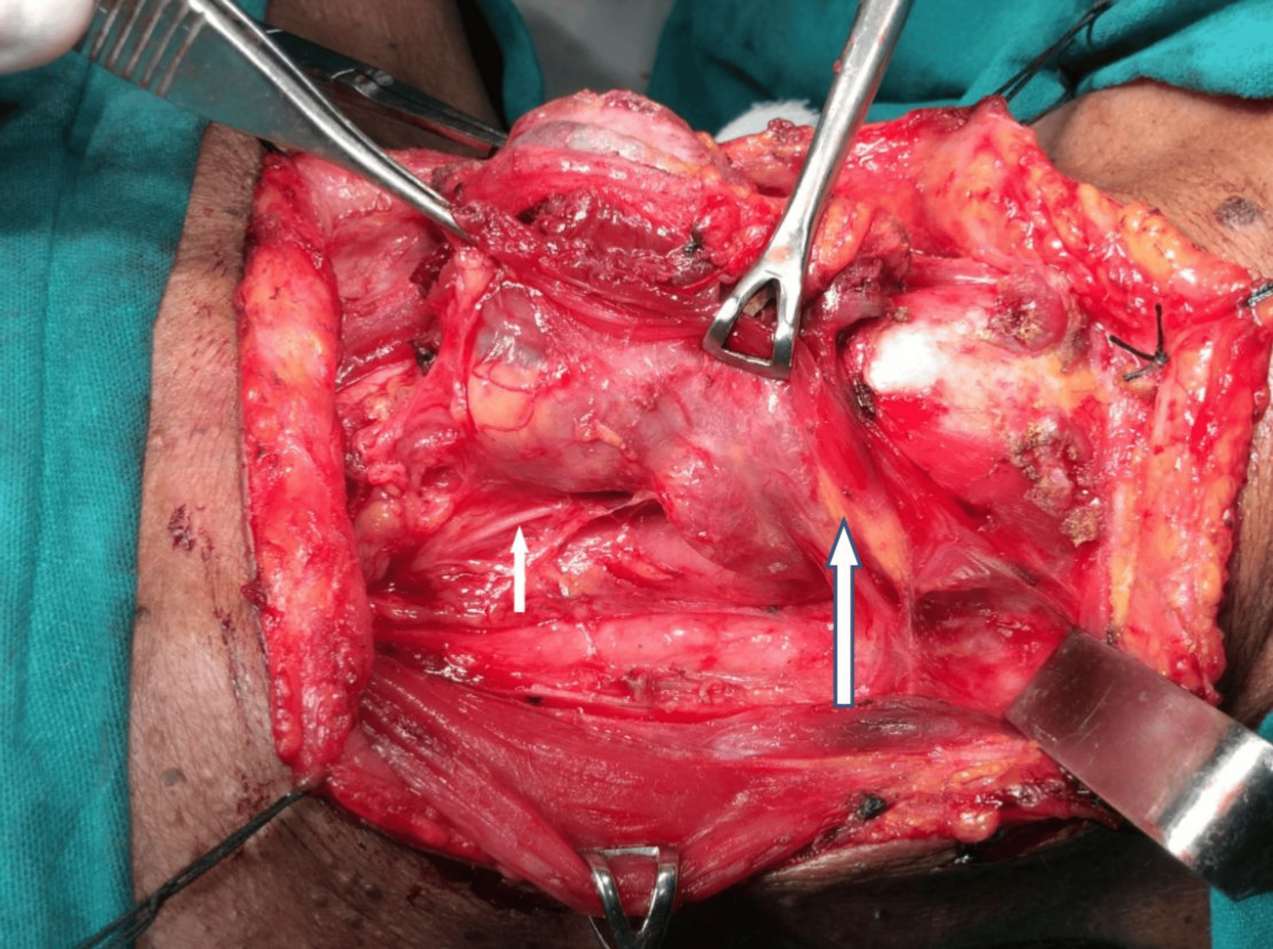
Total thyroidectomy in a 36-year-old woman with thyroid malignancy. White arrows indicate the recurrent laryngeal nerve (short arrow) and the superior parathyroid gland (long arrow).

Figure 3 shows total thyroidectomy being performed in a case of papillary carcinoma thyroid.

**Figure 4:**
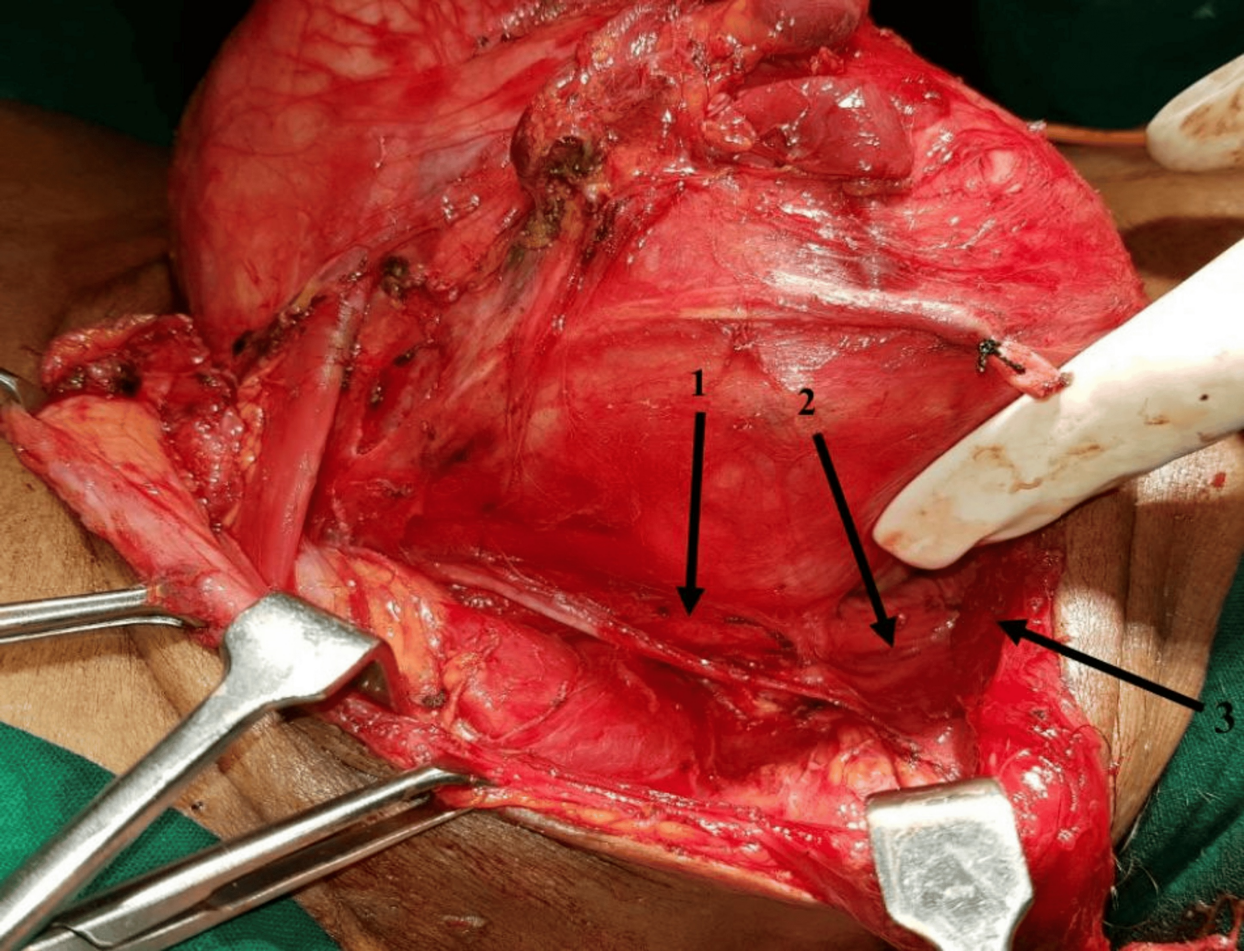
Total thyroidectomy being performed on a 46-year-old woman. 1. Ansa cervicalis; 2. Middle thyroid vein; 3. Internal carotid artery.

Figure 4 shows the procedure of total thyroidectomy, with the tumour being mobilized from the carotid sheath.

Fourteen cases (50%) had a minimal retrosternal extension, extending less than 1 inch below the suprasternal notch, with 21.4% experiencing transient hypocalcemia. Six cases had an extension of 1-2 inches below the suprasternal notch, with 66.6% showing hypocalcemia; of these, four experienced transient hypocalcemia, while two had permanent hypocalcemia. In five cases with extension 2-2.5 inches below the suprasternal notch, 80% experienced hypocalcemia, with two cases being transient and two permanent. One case extended to the superior border of the aortic arch and resulted in permanent postoperative hypocalcemia. Additionally, two cases had retrosternal with retroclavicular extension - one had permanent hypocalcemia, while the other remained normocalcemic.

The odds of hypocalcemia are higher in individuals with retrosternal extension compared to those without (odds ratio = 3.58). Thirteen cases with retrosternal extension developed postoperative hypocalcemia, with 10 of these patients showing serum calcium levels between 8 and 8.3 mg/dL. In two patients, serum calcium levels dropped to 7.2 and 7.8 mg/dL, and in one patient, levels decreased to 6.7 mg/dL.

Table 4 outlines the duration taken for the correction of hypocalcemia among different patient groups. Around 69.15% of hypocalcemic patients with retrosternal extension had transient and 30.7% had permanent hypocalcemia. A total of 87.5% of hypocalcemic patients post-total thyroidectomy had transient and 12.5% had permanent hypocalcemia while 30.7% of hypocalcemic patients post-total thyroidectomy with central compartment clearance had permanent hypocalcemia.

**Table 4:** Duration taken for correction of hypocalcemia

Duration taken for correction of hypocalcemia	Patients with retrosternal extension (n=28); Hypocalcemic patients=13	Patients with no retrosternal extension (n=41); Hypocalcemic patients=8	Patients who underwent total thyroidectomy (n=29); Hypocalcemic patients=9	Patients who underwent total thyroidectomy + central compartment clearance (n=40); Hypocalcemic patients=12
<1 month	6	5	6	5
2-3 months	3	2	2	3
Remained hypocalcemic (>3months)	4	1	1	4

The odds of permanent hypocalcemia are higher in patients with immediate onset compared to those with delayed onset (odds ratio = 1.75). Among the 21 patients with hypocalcemia, 10 showed clinical signs. Five patients with serum calcium levels ranging from 8 to 8.3 mg/dL had a positive Chvostek’s sign. Two patients with calcium levels of 8 and 8.1 mg/dL exhibited a positive Chvostek’s sign along with muscle cramps. Two other patients experienced tingling and numbness in their fingers with serum calcium levels of 7.2 and 7.8 mg/dL. One patient, with a serum calcium level of 6.7 mg/dL, had muscle cramps, tingling sensation in the fingers, and presented as drowsy and confused.

Table 5 provides information on the duration for which patients received calcium supplements after total thyroidectomy in patients with and without retrosternal extension.

**Table 5:** Comparison of duration of calcium supplements recieved by 69 patients after total thyroidectomy with or without retrosternal extension.

Duration of calcium supplements received	Total (n=69)	With retrosternal extension (n=28)	Without retrosternal extension (n=41)
<=3 months	34	10	24
1 year	12	5	7
2 years	5	3	2
Death due to other causes	3	2	1
Lost follow-up	5	2	3
Still continuing to receive calcium	10	6	4

All patients were operated through the neck and none required sternotomy or tracheostomy, and there were no cases of recurrent laryngeal nerve injury. Our study excluded patients needing completion thyroidectomy, as those who had previously undergone lobectomy or hemithyroidectomy at other centres could have had parathyroid injury or devascularization. Two patients on long-term calcium supplements developed renal stones, and one developed ischemic heart disease. Vitamin D deficiency, which can contribute to hypocalcemia, is often associated with limited sun exposure, the use of sunscreens, and protective clothing (e.g., traditional long garments). In our study, 33% of patients wore long garments, 38% were homemakers with limited sunlight exposure, and 28% were farmers who regularly received sunlight exposure.

## Discussion

Total thyroidectomy is a commonly performed procedure in the Department of Otorhinolaryngology and Head and Neck Surgery, often indicated for benign and malignant thyroid conditions. One of the most significant postoperative complications is hypocalcemia, resulting from inadvertent damage or devascularization of the parathyroid glands during surgery. This can lead to transient or permanent hypocalcemia, with symptoms ranging from mild neuromuscular irritability to severe, life-threatening conditions. The risk of hypocalcemia is particularly higher in cases requiring extensive surgical dissection, such as those with retrosternal extension or central compartment clearance.

In our study, 30.4% of patients experienced hypocalcemia after total thyroidectomy, with a higher incidence (odds ratio = 3.58) in those with retrosternal extension. The difference was not statistically significant, but the trend aligns with existing literature, which emphasizes the increased risk of hypocalcemia due to more complex surgical dissection in these cases; however, a study with large numbers may show a statistically significant difference.

Nair et al. (2014) reported hypocalcemia incidence of up to 30% in patients undergoing total thyroidectomy, with higher risks observed in patients who required more extensive surgeries, such as central compartment clearance or retrosternal dissection [15]. This supports our study’s observation that retrosternal extension and complex surgeries increase the risk of devascularization of the parathyroid glands, which is a major cause of hypocalcemia postoperatively.

A study by Nahas et al. (2006) noted that the use of a short-stay protocol for thyroidectomy patients could effectively reduce hospital costs without increasing the risk of severe hypocalcemia in patients with lower surgical complexity [13]. However, for patients requiring more extensive procedures, such as those with retrosternal extension, they recommended more rigorous calcium monitoring. The prolonged hypocalcemia observed in our study, with 42.1% of affected patients requiring more than one month for calcium normalization, is similar to their findings that the complexity of the surgery significantly impacts calcium metabolism recovery time.

Another similar study by Thakur et al. (2023) focused on the role of postoperative serum PTH levels as a predictor of hypocalcemia and found that patients with lower PTH levels shortly after surgery were more likely to develop significant hypocalcemia [16]. Although our study did not measure PTH levels, the relationship between the extent of surgery and postoperative hypocalcemia is similar, suggesting that surgical techniques that preserve parathyroid function may reduce the risk of prolonged hypocalcemia.

In our study, those who had an early drop in serum calcium levels within 48 hours had a higher incidence of permanent hypocalcemia (odds ratio = 1.75) compared to those with late onset. A study conducted by Reid et al. (2015) demonstrated that calcium supplementation is critical during the early postoperative period. This finding is supported by our study, where nearly half of the patients required calcium supplementation for up to three months, and some continued to need it for over two years [4]. The 14.3% incidence of calcium supplementation-related complications, such as renal stones and gastrointestinal issues, mirrors reports in the literature on the long-term risks of calcium supplementation.

In our study, we have highlighted the risk with retrosternal extension or diagnosis of malignancy or central compartment clearance using log regression or odds ratio, which many studies have not addressed. Our study shows that retrosternal extension and central compartment clearance are associated with increased risks of postoperative hypocalcemia. Similar to previous studies, this underscores the need for meticulous surgical techniques to preserve parathyroid function and rigorous postoperative calcium management. These findings, while not statistically significant, highlight a clinically relevant trend that future studies could investigate further.

Limitations

A smaller sample size may fail to detect subtle yet clinically significant differences; therefore, a larger sample size would enhance the statistical significance of the findings. Additionally, the retrospective nature of the study limits control over potential confounding factors. Conducting further prospective studies could provide stronger evidence.

## Conclusions

Our study suggests that retrosternal extension and more extensive surgical procedures increase the risk of postoperative hypocalcemia and prolong its recovery. Although not statistically significant, the observed trends indicate the need for careful intraoperative techniques to preserve parathyroid function. Furthermore, postoperative calcium monitoring and long-term supplementation remain critical for managing patients at higher risk of hypocalcemia. Future prospective studies with larger sample sizes are recommended to further investigate the relationship between surgical complexity and hypocalcemia, enabling better prediction and management of these complications.
